# Machine learning-based prediction of breast cancer growth rate in vivo

**DOI:** 10.1038/s41416-019-0539-x

**Published:** 2019-08-09

**Authors:** Shristi Bhattarai, Sergey Klimov, Mohammed A. Aleskandarany, Helen Burrell, Anthony Wormall, Andrew R. Green, Padmashree Rida, Ian O. Ellis, Remus M. Osan, Emad A. Rakha, Ritu Aneja

**Affiliations:** 1 Department of Biology, Georgia State University, Atlanta, GA 30303 USA; 2 Nottingham Breast Cancer Research Centre, Division of Cancer and Stem Cells, School of Medicine, University of Nottingham and Nottingham University Hospitals NHS Trust, City Hospital Campus, Nottingham, NG5 1PB UK; 3 Nottingham Breast Institute, Nottingham University Hospitals NHS Trust, Nottingham City hospital, Nottingham, NG5 1PB UK; 4 Mathematics and Statistics, Georgia State University, Atlanta, GA 30303 USA

**Keywords:** Breast cancer, Cancer models

## Abstract

**Background:**

Determining the rate of breast cancer (BC) growth in vivo, which can predict prognosis, has remained elusive despite its relevance for treatment, screening recommendations and medicolegal practice. We developed a model that predicts the rate of in vivo tumour growth using a unique study cohort of BC patients who had two serial mammograms wherein the tumour, visible in the diagnostic mammogram, was missed in the first screen.

**Methods:**

A serial mammography-derived in vivo growth rate (*SM-INVIGOR*) index was developed using tumour volumes from two serial mammograms and time interval between measurements. We then developed a machine learning-based surrogate model called *Surr-INVIGOR* using routinely assessed biomarkers to predict in vivo rate of tumour growth and extend the utility of this approach to a larger patient population. *Surr-INVIGOR* was validated using an independent cohort.

**Results:**

*SM-INVIGOR* stratified discovery cohort patients into fast-growing versus slow-growing tumour subgroups, wherein patients with fast-growing tumours experienced poorer BC-specific survival. Our clinically relevant *Surr*-INVIGOR stratified tumours in the discovery cohort and was concordant with *SM-INVIGOR*. In the validation cohort, *Surr-INVIGOR* uncovered significant survival differences between patients with fast-growing and slow-growing tumours.

**Conclusion:**

Our *Surr-INVIGOR* model predicts in vivo BC growth rate during the pre-diagnostic stage and offers several useful applications.

## Background

Breast cancer (BC) is a heterogeneous disease with tumours exhibiting variable morphology, molecular profiles, behaviour and response to therapy. Mounting evidence demonstrates that BC shows variable rates of growth, which has important clinical and medicolegal implications.^1–4^ In vivo growth rate is not only a quantifiable trait of the tumour but can also serve as a tool to plan and evaluate screening programmes, clinical trials or epidemiologic studies. In addition, BC growth rate evaluated using tumour size from mammograms may predict tumour response to chemotherapy, and may help in determining the likely time of tumour initiation and previous tumour size in medicolegal cases.^5–7^ BC growth rate is also associated with prognostic variables, such as lymph node status, stage and vascular invasion;^3,4,8^ however, the prognostic and predictive value of BC growth rate has not been harnessed in routine practice due to the inherent difficulty in its assessment in the short intervals between diagnosis and treatment.

Although the growth rate of BC in vivo is strictly regulated, it appears to be dependent on the balance between several variables including growth fraction (the tumour cells that are proliferating and leading directly to the addition of new tumour cells), the rate of tumour cell loss by apoptosis and/or necrosis, tumour cells’ doubling-time/kinetics, and the surrounding microenvironment including angiogenesis, blood supply and host immune response to the proliferating tumour cells.^9–12^ The complexity of the processes controlling BC growth and the interaction with the tumour microenvironment make assessment and prediction of BC growth rate a challenging task. Therefore, serial imaging of BC at different time points is considered as the best model available for assessing the in vivo growth rate and for determining associations between potential intrinsic growth rate determinants and BC behaviour, including response to therapy.

**Fig. 1:**
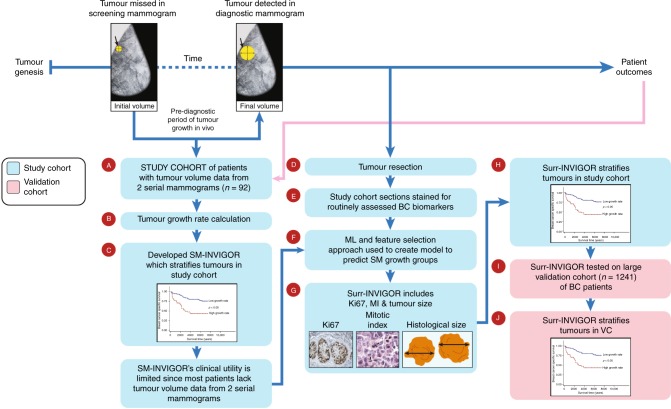
Schematic depicting sequences of steps in our study leading to the calculation of *SM-INVIGOR* and the development of *Surr-INVIGOR* that predicts in vivo tumour growth rate in BC. Briefly, tumour volumes from two serial mammograms and the time interval between measurements in a unique data set of 92 patients (**a**) were used to develop a growth rate index *SM-INVIGOR* (**b**). The growth index significantly predicts BCSS and classifies tumours as slow growing or fast growing (**c**). When the tumours were resected after final diagnosis (**d**), tumour sections were immunohistochemically stained for a panel of BC biomarkers (**e**). A machine-learning algorithm was used to develop a surrogate model (termed *Surr-INVIGOR*) for *SM-INVIGOR* that uses routinely assessed BC clinical biomarkers like Ki67, mitotic index and histological size. The multivariable model non-linearly combines multiple clinicopathological variables and immunohistochemical biomarkers to predict the tumour’s in vivo growth rate prior to diagnosis (**f**, **g**). Using the same growth rate threshold as *SM-INVIGOR*, the *Surr-INVIGOR* model was able to prognostically stratify patients in study cohort (**h**). Finally, *Surr-INVIGOR* was validated using an independent BC validation cohort of 1241 patients and was found to be strongly prognostic in the validation cohort (**i**, **j**)

This study utilises a discovery cohort comprising clinically and molecularly well-characterised data from BC patients who underwent serial mammography. It is a unique and rare cohort because the second mammogram illuminated that the tumour was indeed “missed” during the first mammogram. We find that this one-of-a-kind cohort can be interrogated to (a) identify predictors of BC in vivo growth rate, (b) evaluate the impact of BC growth rate on disease outcome and (c) develop a surrogate model that robustly predicts pre-diagnosis in vivo growth rate for patients who would normally not have tumour volume data from two serial mammograms. In contrast to a matched first-presentation-only BC patients’ cohort, BC growth rate in this study is determined by the changes in tumour volume between sequential mammograms, wherein the first mammogram “mistakenly” reported the case as normal/benign, and the cancer was identified in the screening mammogram on a retrospective review subsequent to the second (diagnostic) mammogram (Fig. 1).

## Methods

### Study cohort

The study cohort comprises 114 BC patients aged between 50 and 70 years who were presented at the Nottingham City Hospital from 1988 to 2008 with BC, and for whom review of the previous screening mammogram showed a previously undetected tumour at the same affected site. This may have been due to either a false-negative screening outcome or due to minimal visible signs of malignancy on the previous mammogram. Mammographic abnormalities included measurable soft tissue abnormality (mass, distortion or asymmetry) on screening and diagnostic films. On retrospective review of the previous mammogram after the disease diagnosis, two radiologists (blinded to each other’s observations) confirmed the “missed” cancer. We selected patients in whom a soft tissue abnormality was detected (upon retrospective review of prior screening mammograms) at the site of the subsequent cancer. Due to a misdiagnosed mammogram, this cohort uniquely comes with an earlier screening measurement with a visible tumour. Clinicopathological data including age, histological tumour type, primary tumour size, lymph node status, histological grade, Nottingham Prognostic Index (NPI), vascular invasion and patients’ outcome data were obtained. BC-specific survival (BCSS) was defined as the time interval (in months) between the primary surgeries and death from BC. The mean survival time of this cohort of patients was 120 months. Clinicopathological variables were available for 92 cases, and the BCSS was available in 90 cases; thus, we restricted our study to these cases (Fig. 1a).

### Calculating tumour volumes and growth rates

The two measurements in the screening and diagnostic mammograms were assumed as tumour diameter and tumour height, which were then used to calculate tumour volumes at the time of screening and diagnosis. The greater mammogram dimension was assumed as height corresponding to the diameter of the semi-major axis, and the other dimension was regarded as diameter of the semi-minor axis. For tumour volume calculation, we considered the aforementioned dimensions as volume inputs for a cylinder, sphere and an oblate spheroid.^13^ For tumour growth rates, we tested exponential growth,^14,15^ the Gompertz model,^16^ and power law growth with the exponent set to both the classic value of 2/3^17,18^ and 1/2^19^ as shown in Table S1. For all models, the initial volume for the growth rate was determined using the screening mammogram, and the final volume was determined from the diagnostic mammogram, with the time variable denoted by the days between the two mammograms.

### Selecting optimal tumour volume, growth rate combination and development of SM-INVIGOR

Multiple tumour volume/three-dimensional shape assumptions and growth rate functions used in previous studies^19^ were tested to find the optimal combination that was prognostic. Growth rate indices that combined tumour volume (calculated assuming the tumour to be a sphere, cylinder or spheroid) and individual growth functions (calculated assuming exponential growth, two sets of the Power Law function (α = 1/2 or 2/3) or Gompertz growth) were compared on the basis of their prognostic ability. Growth rates were used either as a continuous variable or through a fast/slow growth cut-off determined through optimising the log-rank statistic.^20,21^ Both forms of all growth rates were analysed univariately in a Cox proportional hazard regression model using 10-year breast cancer-specific survival (BCSS), and corresponding model fits were ranked with the Akaike Information Criterion (AIC).^22^ The best-fitting growth rate index was chosen via the lowest relative AIC and was used in subsequent analyses. The data related to changes in volume of the lesion between the time of screening and at diagnosis, as well as the time between screening and diagnosis, were used to estimate the **S**erial **M**ammography-derived ***In****-****vi****vo* **G**r**o**wth **R**ate (*SM-INVIGOR*) (Fig. 1b). To control for common clinicopathological confounders, the growth rate model was also analysed with multivariate Cox regression alongside grade, age and oestrogen receptor (ER) status. In addition, the tumour volumes at the screening and diagnostic time points were tested prognostically to evaluate the prognostic significance of the change in tumour volume versus that of the screen- or diagnostic mammogram-calculated volume individually (Fig. 1c).

### Assessing and scoring immunohistochemical staining

For each patient, a representative formalin-fixed paraffin wax-embedded (FFPE) tumour block of the resected tumour was obtained from the Nottingham breast tumour bank (Fig. 1d). Full-face sections 4-μm thick from the representative FFPE tumour blocks were prepared onto Xtra® Surgipath glass slides, and were used for immunohistochemical (IHC) assessment of the following markers: ER, progesterone receptor (PR), human epidermal growth factor receptor 2 (HER2), the proliferation markers Ki67 and MCM2 (minichromosome maintenance 2), the basal markers CK5/6 (cytokeratin 5/6) and epidermal growth factor receptor (EGFR), the apoptosis markers BCL2 and cleaved caspase-3. IHC was performed on tissue sections using the Novolink™ Max Polymer Detection System (Leica, Newcastle, UK). Briefly, heat-assisted retrieval of antigen epitopes was performed in citrate buffer (pH 6) using a microwave for 20 min, followed by immediate cooling. The slides were rinsed with Tris-buffered saline (TBS, pH 7.6). The primary antibodies as summarised in Table S2 were applied for 30 min at room temperature, except for cleaved caspase-3 staining. For cleaved caspase-3, a pre-fabricated detection kit (*SignalStain®* *Cleaved Caspase-3 (Asp175) IHC Detection Kit #8120, Cell Signaling Technology)* was used following the manufacturer’s instructions. Other markers were stained using our protocols as previously published.^23,24^

Appropriate positive and negative controls were used for each marker and included in each staining run. Only the invasive tumour cells were scored independently by two observers (SB and MA) blinded to each other’s scores and clinicopathological data. Cases with discordant results were further reviewed by both observers to achieve scoring consensus. For each marker, the percent and intensity of staining were assessed, and H-scores were generated. For ER, PR and HER2, cut-offs according to published guidelines were used.^25,26^ Ki67 and cleaved caspase-3 were assessed and scored as previously described.^23,24^ BC molecular subtypes were defined based on their IHC expression profile into (a) luminal (ER + and/or PR + /HER2-), (b) HER2 + (HER2-positive), (c) triple-negative (TN; ER-, PR-, HER2-) and (d) basal-like breast cancer (BLBC: TN + CK5/6 + ).^24^ A total of 92 cases were informative for IHC biomarkers, and these comprised the study cohort in the subsequent analyses including molecular markers (Fig. 1e).

### Development of the machine learning-based surrogate model (Surr-INVIGOR)

The above-mentioned clinical and molecular variables and immunohistochemical biomarkers (Table S3) were evaluated using machine-learning algorithms to identify an optimal feature set that could serve as a surrogate model for *SM-INVIGOR* to predict fast or slow in vivo growth rate for cases where only a single (diagnostic) mammogram is available (Fig. 1f–h). The significance of mean differences for all potential surrogate variables, between fast-growing and slow-growing tumours, was first calculated using a two-tailed *t* test; this was followed by a ranking of the variables based upon their discriminating capacity. Multiple classification algorithms (support vector machines, naive Bayes, decision trees, discriminant analysis and ensemble) with optimised hyperparameters^27,28^ were then tested. The machine-learning algorithm and feature set that resulted in the maximum fivefold cross-validated accuracy (mean of 100 iterations) were chosen. For each trained machine-learning model (combination of biomarkers), hyperparameters were fit through Bayesian optimisation^27,28^ over 180 iterations (Table S4). Furthermore, a combination of variables was used, in an optimised regression model, to identify if the continuous growth rate value for each patient could be determined. Finally, the outputs from the machine learning-based approach were compared with the regression-based models, which did not yield good R^2^ values owing to small sample size.

### Validation of Surr-INVIGOR

The prognostic performance of this surrogate model (*Surr-INVIGOR*) was tested in an independent, well-characterised large validation cohort of 1241 BC patients using Kaplan–Meier survival analysis (Fig. 1i, j). Multivariate Cox regression was used to control for confounding effects of common clinicopathological variables.

### Statistical analysis

All statistical analyses were carried out with SAS 9.4® software and MATLAB version 9.2. Clinicopathological proportion differences between growth groups were determined using the χ^2^ test. Continuous clinicopathological variable differences were evaluated via a two-tailed *t* test. Prognostic time to event analysis was performed using Kaplan–Meier and Cox proportional hazard regression, wherein a death due to BC was considered as an event and every other outcome was censored. For all analyses, *p* < 0.05 was considered significant.

## Results

### Clinicopathological and molecular features of cases in the study cohort

Most patients in the study cohort showed features associated with good prognosis, including lower grade and negative (65%) or early positive (pN1; 26%) lymph nodes. Age at the time of diagnosis ranged from 50 to 73 years (mean = 60.3 years, median = 61.0 years). There was a predominance of the luminal A subtype with 85% positive for ER, while HER2 overexpression was identified in only 6% of the patients. Ki67 staining ranged from 0 to 96%, with a mean expression of 19% (Table 1). Moreover, there was a significant correlation between the histological tumour size and the mammogram tumour size at time of diagnosis (Pearson’s correlation = 0.58870; *p* < 0.0001).

**Table 1:** Clinicopathological characteristics of cases in the study cohort and validation cohort

Parameters	Study cohort	Validation cohort
	Number of cases (*N*; %)	Number of cases (*N*; %)
Age		
≤65	75 (81.5)	1057 (85.2)
>65	17 (18.5)	184 (14.8)
Tumour grade		
1	16 (17.4)	325 (26.2)
2	42 (45.7)	501 (40.4)
3	34 (36.9)	415 (33.4)
Tumour size		
≤15	32 (35.0)	969 (72.31)
>15	60 (65.0)	371 (27.69)
Lymph node		
1	60 (65.2)	763 (61.5)
2	24 (26.1)	382 (30.8)
3	8 (8.7)	96 (7.7)
Hormone receptor status		
ER positive	78 (84.8)	915 (73.7)
ER negative	14 (15.2)	326 (26.3)
PR positive	59 (64.1)	675 (54.4)
PR negative	33 (35.9)	566 (45.6)
HER2 expression		
Positive	5 (5.4)	151 (12.2)
Negative	81 (88.0)	1058 (85.3)
Missing	6 (6.5)	32 (2.6)
Intrinsic molecular subtypes		
Luminal A	38 (41.3)	408 (32.9)
Luminal B	28 (30.4)	429 (34.6)
HER2	5 (5.4)	151 (12.2)
BLBC	4 (4.3)	138 (11.1)
Triple negative	11 (12.0)	68 (5.5)
Missing	6 (6.5)	47 (3.8)
Ki67		
High	44 (47.8)	667 (53.7)
Low	48 (52.2)	574 (46.3)
Tumour type		
Invasive no special type	50 (54.3)	761 (61.3)
Invasive lobular	17 (18.5)	93 (7.5)
Tubular	11 (12.0)	299 (24.1)
Mucinous	2 (2.2)	11 (0.8)
Mixed type	12 (13.0)	77 (6.2)
Coexisting DCIS		
None	21 (23.0)	NA
Low grade	20 (22.0)	NA
Intermediate grade	22 (24.0)	NA
High grade	29 (31.0)	NA
Lymphovascular invasion		
Negative	60 (66.2)	686 (55.3)
Definite	21 (22.8)	397 (32.0)
Probable	11 (11)	158 (12.7)
Outcome status		
Alive	62 (67.4)	650 (52.3)
Dead	30 (32.6)	591 (47.6)

### Development of SM-INVIGOR, a significant predictor of BCSS

Since fast in vivo growth prior to diagnosis is a sign of aggressive disease and could lead to poor outcomes, we reasoned that the growth rate model of choice would be the one that is most prognostic. Thus, we evaluated various combinations of growth rate functions and assumptions regarding the tumour’s three-dimensional shape. The best-fitting model of tumour volume and growth rate was obtained using the assumption that the study cohort comprises spherical tumours growing at a power law (α = 0.5) rate; this growth rate function (*SM-INVIGOR)* stratified the tumours into slow-growing and fast-growing subgroups and produced a minimum cross-validated AIC of 152.621 (Table S5). Using these assumptions, tumour volumes at the time of screening ranged from 53 to 56,115 mm^3^ (mean of 2742 ± 7619 mm^3^). This contrasted with tumour volumes at diagnosis, which ranged from 61 to 61,562 mm^3^ (mean = 5573 ± 8768 mm^3^). The mean time difference between date of first screening and that of second diagnostic screening was 18 months (range 4–37 months, median = 17.5 months). Tumour growth rate differed considerably from patient to patient, ranging from 0 to 0.53 mm^3^/day (mean = 0.08 ± 0.13 mm^3^).

**Fig. 2:**
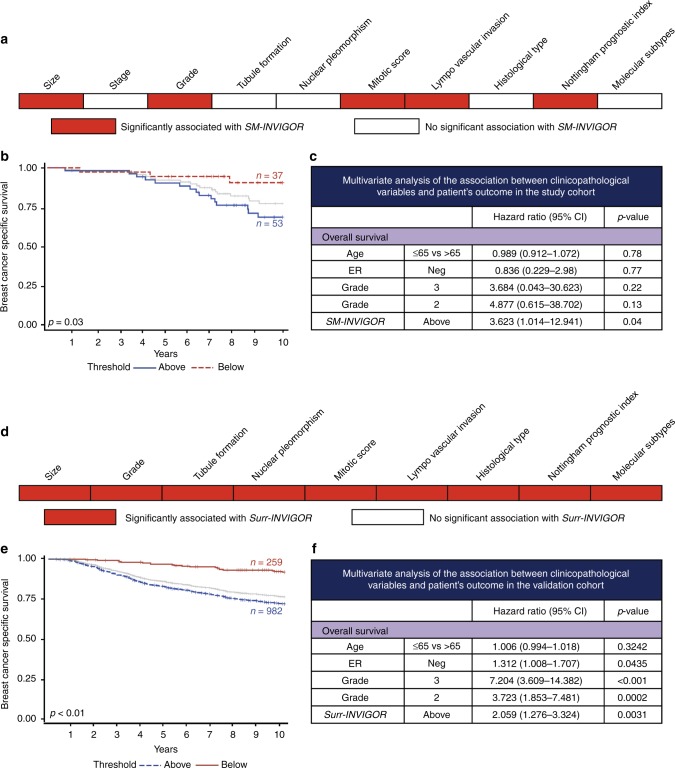
Prognostic significance of *SM-INVIGOR*. **a** Univariate associations between clinicopathological parameters and *SM-INVIGOR*. **b** Kaplan–Meier survival curve for study cohort patients stratified into high and low growth rate groups by SM-INVIGOR. **c** Multivariable analysis of the association between clinicopathological variables and outcome [breast cancer-specific survival (BCCS)] in study cohort. **d** Univariate association between clinicopathological parameters and *Surr-INVIGOR* in validation cohort. **e** Kaplan–Meier survival curve for patients stratified into high and low growth rate subgroups by *Surr-INVIGOR* in validation cohort. **f** Multivariable analysis of the association between clinicopathological variables and BCSS in validation cohort

*SM-INVIGOR* used a cut-off of 0.045 mm^3^/day to stratify tumours into slow-growing (*n* = 53) and fast-growing (*n* = 37) subgroups. Faster *SM-INVIGOR* significantly associated with clinicopathological factors normally associated with poorer prognoses, such as larger histological tumour size (*p* = 0.0023), high grade (Grade 3) (*p* = 0.0186), more mitotic divisions (*p* = 0.0134), apparent vascular invasion (*p* = 0.0139) and a poor NPI (*p* = 0.011) (Fig. 2a). *SM-INVIGOR* varied significantly between BC molecular subtypes with the highest rate observed in triple-negative BC (TNBC) compared with other subtypes (*p* < 0.05). Among the proliferation/apoptosis-related biomarkers that were immunohistochemically assessed (Table S3), only Ki67 showed a significant mean difference (*p* = 0.0003) between the fast-growing (24%) versus slow-growing (11%) tumour subgroups. Furthermore, patients with higher tumour growth rate showed significantly poorer survival (BCSS = 71.7%) relative to the slow-growing tumours (BCSS = 91.9%) as shown in Kaplan–Meier’s survival graph (Fig. 2b). *SM-INVIGOR* retained prognostic significance (*p* = 0.0299, high growth rate HR = 4.605) upon controlling for common clinicopathological variables, including grade, age and ER status. In fact, *SM-INVIGOR* was the only variable significantly associated with BCSS in our multivariable analysis (Fig. 2c).

### Development of a clinically relevant surrogate model (Surr-INVIGOR) for in vivo growth rate prediction

Unlike the patients in our unique discovery cohort, most begin therapy at an initial cancer diagnosis, and are therefore unlikely to have two serial mammograms with two tumour volume measurements. Because of this difference, *SM-INVIGOR* is limited in its utility to derive in vivo tumour growth rate for most BC patients in routine clinical practice. Therefore, to extend the benefits of having growth rate data (or estimates) to a much larger group of patients lacking a second mammogram, we developed a machine learning-based surrogate growth rate model for *SM-INVIGOR* and called it *Surr-INVIGOR* (described in Supplementary Data). *Surr-INVIGOR* non-linearly combines multiple clinicopathological variables and immunohistochemical biomarkers to predict in vivo growth rate. First, we evaluated the ability of individual clinicopathological variables to serve as potential surrogate features and discriminate between the fast-growing and slow-growing tumour subgroups of our study cohort (*p*-values for mean difference between the subgroups is shown in Table S4). Ki67 (*p* = 0.000265), mitotic score (MI; *p* = 0.002479), tumour size (*p* = 0.003619), NPI (*p* = 0.004163) and grade (*p* = 0.021128) differed significantly between the fast-growing and slow-growing tumours. The seven variables (Ki67, mitotic score, tumour size, NPI, grade, stage and tumour size) with *p-*value < 0.2 were then tested in multiple machine learning-based classification algorithms via sequential selection (Fig. S1). The maximised cross-validated accuracy, which indicates the optimal *Surr-INVIGOR* model, was obtained when three features (Ki67, MI and histological tumour size) were used in a K-nearest neighbour algorithm or KNN (accuracy or concordance with the classification yielded by *SM-INVIGOR* = 0.706). The ensemble also yielded a 70% accurate classifier, but required four additional features; the more parsimonious KNN was thus selected for use in *Surr-INVIGOR*. Fitting an optimal regression model to predict the growth rate continuously resulted in a poor R^2^, peaking at 0.22, as shown in Fig. S2, perhaps owing to the small sample size. Thus, our machine learning-based *Surr-INVIGOR* model was a clinically relevant, superior choice compared with regression-based models.

### Validation of *Surr-INVIGOR* in an independent BC case series demonstrates its robust prognostic value

We then evaluated the prognostic ability of *Surr-INVIGOR* in an independent BC case series (*n* = 1241) from Nottingham University Hospital, UK. Patient age at the time of diagnosis ranged from 21–71 years (mean = 53.6 years, median = 54 years). Most patients showed features associated with good prognosis, including negative lymphovascular invasion (55.3%), and negative (61%) or showed 1–3 positive (30%) lymph nodes. Patient follow-up time ranged from 1 to 120 months (mean = 100.237, median = 120). The clinicopathological features of patients are summarised in Table 1.

The clinicopathological variables that discriminated between slow-growing and fast-growing tumours are depicted in Fig. 2d. Applying the previously trained *Surr-INVIGOR* model, using the same input parameters on this naive validation cohort resulted in significant BCSS stratification. Patients in the fast growth rate group (*n* = 922, BCSS = 72.9%) had a significantly lower survival than patients in the slow growth rate group (*n* = 269, BCSS = 92.3%; Fig. 2e). After accounting for potential clinicopathological cofounders, *Surr-INVIGOR* retained prognostic significance (HR = 1.758, *p* = 0.0361) alongside grade as shown in Fig. 2f.

### *Surr-INVIGOR* can be used to determine tumour age at diagnosis in a subset of breast tumours

Using the different growth rate groups, we can estimate tumour age and the time of inception of a subset of tumours. Assuming the highest (bounded) power law (α = 0.5) growth rate (0.04593 mm^3^/day) for the slow-growing subgroup, we can estimate the date after which the tumour was definitely present within the patients in the slow-growing tumour subgroup. Using these assumptions, we determined that the average tumour age at diagnosis of slow-growing tumours was 4.7 years (Fig. S3). Using this methodology, it may be possible to determine whether a patient possessing a slow-growing tumour undetected at earlier screenings had received a true-negative or false-negative (i.e., tumour was missed) screening result.

## Discussion

Although several studies have investigated variables associated with pre-diagnosis in vivo BC growth rate, only clinicopathological variables and a few molecular biomarkers have been studied in this context, and the available tumour dimensions were limited due to the measurement of the tumour’s long-axis only.^2,5,29,30^ This study utilised a unique cohort of cases with tumour volume measurements (derived using tumour diameter and height data) available from a pair of serial mammograms to derive their in vivo growth rates (*SM-INVIGOR*). We explored the potential association of a larger number of molecular biomarkers with their in vivo BC growth rate, reaffirmed that fast tumour growth rate has a profound impact on prognosis, developed and validated a surrogate model (*Surr-INVIGOR*) that can predict a gross scale (fast versus slow) in vivo growth rate accurately in routine practice, and its medicolegal consequences.

The success of breast screening lies in the timely detection of cancer on mammography. False-negative mammography is among the principal reasons for delayed diagnosis of BC.^31–34^ Even though some authors quote high sensitivity (>90%) for diagnostic mammography, such results are not universal.^35^ Among many factors, age appears to be one of the important factors underlying false-negative reporting, because the high-radiographic density of breast in young women makes detection difficult.^6^ Mammograms are generally capable of detecting tumours as small as 2 mm in diameter, which equates to a tumour of ~10^7^ cells and about 23 tumour doublings.^36^ In our study cohort, however, patients with tumours ranging from 4 to 55 mm received false-negative diagnoses in their screening mammograms showing the imperfection associated with this technology and inherent human limitations associated with reading radiology films. Whether the spread of a tumour is due to delays in diagnosis and initiation of treatment, or due to the inherently more aggressive nature of the tumour cells themselves (i.e., higher in vivo tumour growth rate) is another highly controversial matter. Natural fears that the delay in diagnosis has reduced their chances of survival or of avoiding the life-sapping effects of chemotherapy, or the feeling that cosmetic outcomes which would have been better had the tumour been detected earlier, are frequent causes of patients seeking legal redress. The importance of breast imaging in BC diagnosis and the use of mammography in screening has thus pushed breast radiologists into the frontline for medicolegal actions.^37^ Cancers missed at screening but followed by a positive diagnostic mammogram are not common, yet false-negative mammography is amongst the principal reasons for delayed diagnosis of BC.^31–34^ Only few population-screening programmes have reported the data on this group of cancers, which makes our study cohort uniquely valuable. This cohort allowed us to develop a model to predict pre-diagnostic in vivo tumour growth rate, and provide insights into the potential prognostic consequences of delays in BC diagnosis.

Our study has yielded several key insights into features and the prognostic significance of the rate of tumour growth in its early stages. In our study, we found that *SM-INVIGOR* varies considerably, and is consistent with findings by Weedon-Fekjaer et al.^5^ who reported that the time BC takes to grow from 10 mm to 20 mm in diameter varied from less than 1.2 months to more than 6.3 years. Our current study also reinforced previous findings that higher grade and larger tumours with high proliferative activity are likely to have faster *SM-INVIGOR*, and that faster pre-diagnosis growth rate predicted shorter survival.^2,5,29,30,38,39^ We also found that the status of lymphovascular invasion (LVI) correlated with growth rate; with highly proliferative and fast-growing tumours more likely to develop when there is increased provision of nutrients to the tumour cells from the leaky invaded blood vessels. Our results indicated that increasing *SM-INVIGOR* increases the risk of mortality of the disease. However, *SM-INVIGOR* cannot be included as a prognostic variable in routine clinical practice because of difficulty in evaluating it in the short interval between diagnosis and treatment.

Therefore, we developed *Surr-INVIGOR* to predict the pre-diagnosis in vivo BC growth rate after testing multiple clinicopathological and molecular variables (individually and in combination) using diverse machine-learning algorithms. The optimal algorithm, a KNN which used Ki67, MI and size, stratified both the study and validation cohorts into two subgroups with very distinct outcomes. *Surr-INVIGOR* further allowed routine clinical parameters to be used in patients with slow-growing tumours to determine tumour size at various time points before the diagnosis of the tumour. For fast-growing tumours, immediate surgery is often recommended, as delays may result in upgrading of clinical T stage. *Surr-INVIGOR* may thus have a potential use in medicolegal cases, and may be used to guide screening and perhaps even follow-up intervals in selected groups of BC patients.

Consistent with previous studies,^40,41^ the results from our validation cohort showed a significant correlation between BC molecular subtypes and pre-diagnosis tumour growth rate, wherein a higher growth rate was observed in triple-negative/basal-like BC patients. Previous studies have indicated that faster-growing tumours lead to poorer survival.^42–45^ Our results compellingly demonstrated that high pre-diagnosis in vivo BC growth rate increases the risk of mortality from the disease regardless of potential clinicopathological cofounders. Some previous studies did not find such statistically significant associations,^3,4^ which might be because in those studies, the tumour volume was calculated using only one dimension—a method that can introduce considerable inaccuracy into growth rate calculations. In this study, we utilised a combination of power law growth rate and spherical volume, both of which were significant in a previous study using two-dimensional breast mammogram data,^19^ and showed the most significant prognostic relevance in our data.

Review of previous mammography is carried out as a routine practice at Nottingham Hospital, and cases that show an abnormality at the same site as the diagnosed tumours are considered as cancers potentially missed in the prior screening. Some of these tumours are only detectable in retrospect with knowledge of the diagnostic mammograms, and if all such subtle areas were recalled for further assessment, this would likely increase the false-positive rate beyond what is regarded as acceptable in the NHS breast screening programme. The impact of such delay in the diagnosis on the presentation and outcome of these tumours compared with matched population of women who presented for the first time as symptomatic or with screen-detected BC remains to be defined. Most tumours included in our study (similar to other studies looking at screen-detected tumours) by their very nature, were small, slow-growing luminal tumours and infrequently expressed basal markers or HER2 with similar nodal status.^30^ This can be explained by the unique nature of these slow-growing early-stage tumours in this study. By contrast, aggressive tumours are likely to present without prior mammographic abnormality.^46^ In line with these results, Kalager et al.^47^ have reported that BCs presenting as interval cancers were slightly larger than symptomatic BC, but there was no difference between the two groups regarding lymph node status or patient outcome. Moreover, our results indicated that the impact of *SM-INVIGOR* on disease stage and development of LVI is limited. However, this study holds a few limitations: due to the unique nature of the study cohort and the lack of similar missed cancer cohorts, the *SM-INVIGOR* growth index could not be readily validated. In addition, this is a retrospective, single centre study and adjuvant treatment regimens were not factored in our analyses. Validation of the model in diverse cohorts is necessary before it can be applied for the prediction of in vivo growth rate and determination of the likely tumour initiation date and previous tumour size in clinico-legal cases. If validated in further studies, the model developed herein could potentially guide treatment selection, as it prognostically distinguishes fast-growing tumours from slow-growing ones. For example, for fast-growing tumours, immediate treatment in the form of primary systemic therapy (rather than surgery) may be required. Moreover, HER2 is known to be related to rapid growth of tumours, and might be a good marker to add to the *Surr-INVIGOR*; however, our study cohort was overwhelmingly HER2 negative and thus its impact within a prognostic model could not be properly measured. Further analysis may be required in a diverse cohort.

In conclusion, this study has demonstrated that multiple factors control BC growth; when considered together Ki67, mitotic index and tumour size produce a robust prediction model of pre-diagnostic growth rate and can be used to classify BCs as slow growing or fast growing. The impact of missing subtle cancers in screening mammography seems to depend on whether the tumour was slow growing or fast growing prior to diagnosis, as fast-growing tumours were associated with poorer outcomes and perhaps reflected more aggressive tumour biology. Independent validation of these findings in multiple and more diverse cohorts is warranted.

## Supplementary information

Supplementary files

## Data Availability

All the data and results generated during the study can be provided upon journal request.

## Data availability

All the data and results generated during the study can be provided upon journal request.
