# Supplementary material for: Machine learning-based prediction of breast cancer growth rate in vivo
Source: Br J Cancer. 2019 Aug 9;121(6):497–504. doi: 10.1038/s41416-019-0539-x (PMC6738119; doi:10.1038/s41416-019-0539-x)
Supplement: Supplementary file 1 — Supplementary files [file 41416_2019_539_MOESM1_ESM.pdf]

Supplementary Figures and Tables

**Table S1: Growth rate functions evaluated for development of *SM-INVIGOR***	
**Growth Rate**	**Formula**
Exponential	$Rate = \frac{\log(Volume_{Diagnosis}) - \log(Volume_{Screening})}{Time\ Between\ Diagnosis\ and\ Screening}$
Power Law (α = 2/3)	$Rate = \frac{Volume_{Diagnosis}^{(1-2/3)} - Volume_{Screening}^{(1-2/3)}}{(1-2/3) * Time\ Between\ Diagnosis\ and\ Screening}$
Gompertz	$Rate = \frac{\log(\log\left(10^6 mm^3 / Volume_{Diagnosis}\right)) - \log((\log\left(10^6 mm^3 / Volume_{Screening}\right))}{Time\ Between\ Diagnosis\ and\ Screening}$
Power Law (α = 1/2)	$Rate = \frac{Volume_{Diagnosis}^{(1-1/2)} - Volume_{Screening}^{(1-1/2)}}{(1-1/2) * Time\ Between\ Diagnosis\ and\ Screening}$

Table S1: Table showing the mathematical representation of the growth rates tested.

Table S2: Details of Antibodies used in Immunohistochemistry				
**Antibody**	**Clone**	**Raised in**	**Monoclonal or Polyclonal**	**Dilution**
ER α	1D5	Mouse	Monoclonal	1:100
PR	636	Mouse	Monoclonal	1:100
HER-2 (cerb-2)	A0485	Rabbit	Polyclonal	1:100
Ki67	MIB-1	Mouse	Monoclonal	1:100
CK 5/6	D5/16 B4	Mouse	Monoclonal	1:100
MCM2	HPA031496	Rabbit	Polyclonal	1:100
BCl2	124	Mouse	Monoclonal	1:100
EGFR	C31G7	Mouse	Monoclonal	1:50

Table S2: Details of antibodies used and dilutions.

Table S3: Potential surrogate markers for Growth model	
**Variables**	**P-values**
Ki-67	**0.000265**
Mitosis	**0.002479**
Tumor size	**0.003619**
Nottingham prognostic Index	0.004163
Histologic Grade	0.021126
Tumor Stage	0.143553
Tubule formation	0.195234
Cleaved-caspase3	0.205719
BCL2	0.221934
Patient Age	0.226801
Estrogen Receptor	0.270581
MCM2	0.380132
Progesterone Receptor	0.573077
EGFR	0.657271

Table S3: The list of surrogate markers to develop *Surr-INVIGOR*. Ki67, Mitosis and Histological tumor size served as the significant surrogate variables.

Table S4: K Nearest Neighbor (KNN) hyperparameters optimized for *Surr-INVIGOR*				
**Number of Neighbors**	**Distance**	**Distance Weight**	**Exponent**	**Standardized**
30	Standardized Euclidean distance	Inverse	None	FALSE

Table S4: Table with the chosen hyperparameters for the optimal KNN model.

Table S5: Table showing model fit statistics (Akaike information criterion)		
**Growth Rate**	**Volumes**	**AIC cat. BCSS**
**Exponential**	**Sphere**	153.275
	**Cylinder**	155.37
	**Spheroid**	155.592
**Power Law (α =2/3)**	**Sphere**	155.598
	**Cylinder**	155.057
	**Spheroid**	155.243
**Gompertz**	**Sphere**	155.583
	**Cylinder**	155.263
	**Spheroid**	155.627
**Power Law (α =1/2)**	**Sphere**	152.621
	**Cylinder**	155.179
	**Spheroid**	155.306

Table S5: Table showing model fit statistics (Akaike information criterion) for various combinations of growth rate functions and tumor volumes, wherein the growth rate was treated as a categorical variable. The model with the best fit was the one with the lowest AIC (Power Law with an α of 1/2 when used categorically).

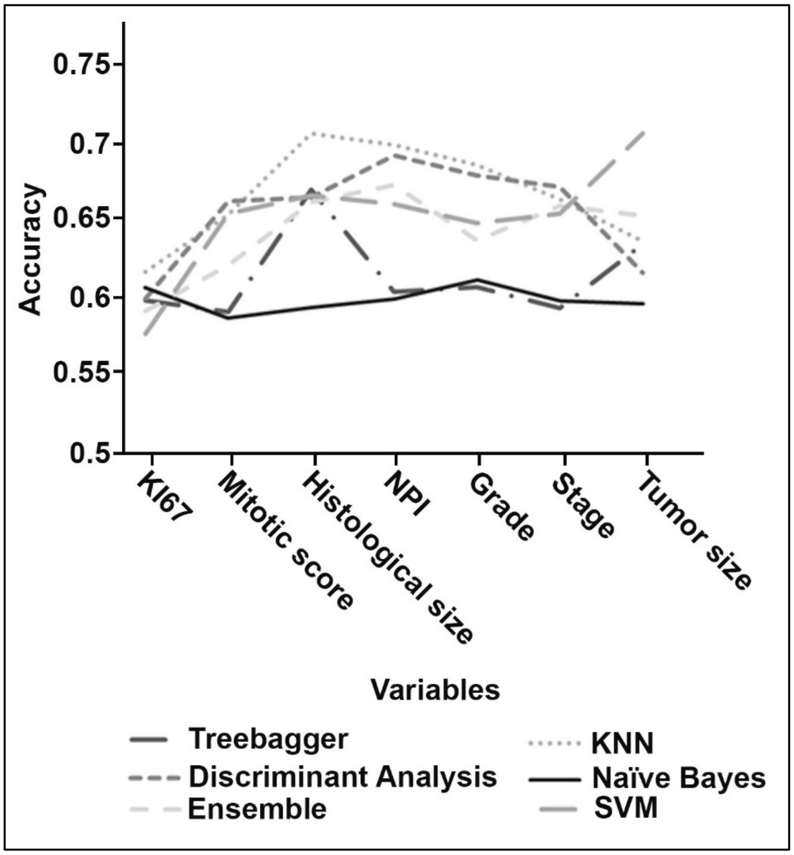

Figure S1: Tumor Growth Rate group (fast versus slow) classification accuracies obtained using different machine learning algorithms and features (included sequentially as indicated on the x-axis). The y-axis represents the cross-validated model performance (accuracy) obtained with inclusion of the indicated discriminatory biomarker and all the ones on its left. Optimal accuracy (~70%) is found using three markers (Ki67, MI, and size) within the KNN algorithm or all 7 variables with the Ensemble algorithm. The KNN was chosen as it minimized the features needed.

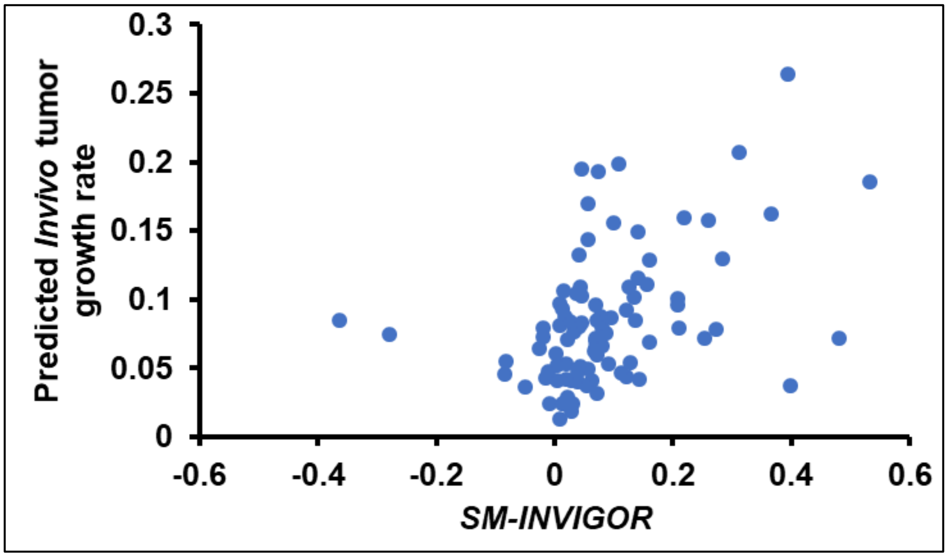

Figure S2: Scatterplot representing the optimal predicted in-vivo growth rate (through regression using surrogate markers) versus the *SM-INVIGOR* from the study cohort. Each dot represents a patient and the calculated R^2 =0.22.

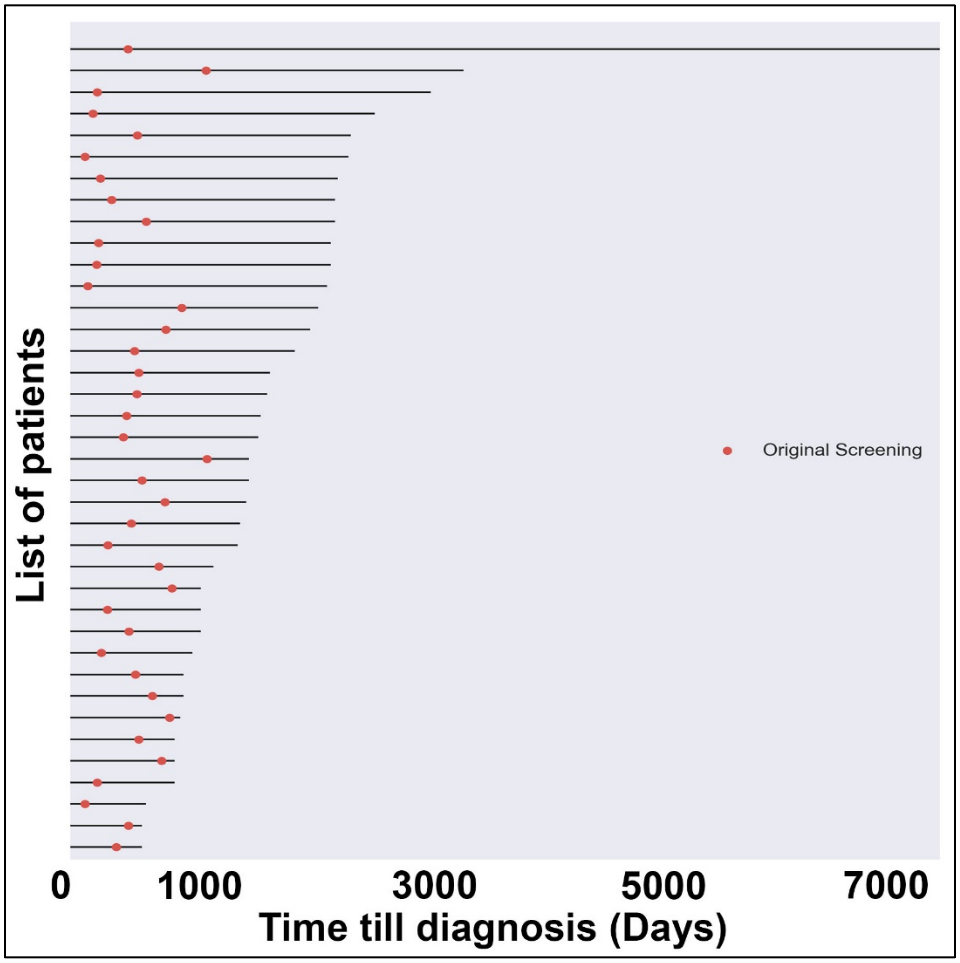

Figure S3: Plot representing the duration before diagnosis that patients in the slow growth subgroup have had a tumor volume >0 mm^3 (black lines). The end of black line represents time of tumor diagnosis. The red points indicate when the original screening was performed. As the growth rate used was the highest found in the slow-growing subgroup, it represented the time that a tumor was conservatively present.
